# Effect of konjac glucomannan on gut microbiota from hyperuricemia subjects *in vitro*: fermentation characteristics and inhibitory xanthine oxidase activity

**DOI:** 10.3389/fnut.2024.1465940

**Published:** 2024-09-19

**Authors:** Jie Deng, Kai Zhou, Caimin Feng, Yilu Bao, Zhiming Zhang, Wenfeng Luo, Meiying Li

**Affiliations:** ^1^Shunde Vocational and Technical College, Foshan, China; ^2^Institute of Jiangxi Oil-Tea Camellia, College of Pharmacy and Life Science, Jiujiang University, Jiujiang, China; ^3^Central Laboratory of Panyu Central Hospital, The Affiliated Panyu Central Hospital of Guangzhou Medical University, Guangzhou, China; ^4^Guangdong Provincial Key Lab of Food Safety and Quality, South China Agricultural University, Guangzhou, China

**Keywords:** konjac glucomannan, short-chain fatty acids, xanthine oxidase activity, hyperuricemia, uric acid

## Abstract

**Background:**

The disorder of uric acid metabolism is closely associated with gut microbiota and short-chain fatty acids (SCFAs) dysregulation, but the biological mechanism is unclear, limiting the development of uric acid-lowering active polysaccharides. Konjac glucomannan (KGM) could attenuate metabolic disturbance of uric acid and modulate the gut microbiota. However, the relationship between uric acid metabolism and gut microbiota is still unknown.

**Methods:**

In this study, The fecal samples were provided by healthy volunteers and hyperuricemia (HUA) patients. Fecal samples from healthy volunteers was regarded as the NOR group. Similarly, 10% HUA fecal suspension was named as the HUA group. Then, fecal supernatant was inoculated into a growth basal medium containing glucose or KGM, and healthy fecal samples were designated as the NOR-GLU and NOR-KGM groups, while HUA fecal samples were designated as the HUA-GLU and HUA-KGM groups. All samples were cultured in an anaerobic bag system. After fermentation for 24 h, the samples were collected for further analysis of composition of intestinal microbiota, SCFAs concentration and XOD enzyme activity.

**Results:**

The results showed that KGM could be utilized and degraded by the gut microbiota from HUA subjects, and it could modulate the composition and structure of their HUA gut microbiota to more closely resemble that of a healthy group. In addition, KGM showed a superior modulated effect on HUA gut microbiota by increasing *Megasphaera*, *Faecalibacterium*, *Lachnoclostridium*, *Lachnospiraceae*, *Anaerostipes*, and *Ruminococcus* levels and decreasing *Butyricicoccus*, *Eisenbergiella*, and *Enterococcus* levels. Furthermore, the fermentation solution of KGM showed an inhibitory effect on xanthine oxidase (XOD) enzyme activity, which might be due to metabolites such as SCFAs.

**Conclusion:**

In conclusion, the effect of KGM on hyperuricemia subjects was investigated based on the gut microbiota *in vitro*. In the present study. It was found that KGM could be metabolized into SCFAs by HUA gut microbiota. Furthermore, KGM could modulate the structure of HUA gut microbiota. At the genus level, KGM could decrease the relative abundances of *Butyricicoccus*, *Eisenbergiella*, and *Enterococcus*, while *Lachnoclostridium* and *Lachnospiraceae* in HUA gut microbiota were significantly increased by the addition of KGM. The metabolites of gut microbiota, such as SCFAs, might be responsible for the inhibition of XOD activity. Thus, KGM exhibited a superior probiotic function on the HUA gut microbiota, which is expected as a promising candidate for remodeling the HUA gut microbiota.

## 1 Introduction

Based on the findings of the Multidisciplinary Expert Consensus on the Diagnosis and Treatment of Hyperuricemia-Related Diseases in China (2023 Edition), it is reported that HUA has a prevalence rate of 13.3% in China, making it the second most prevalent metabolic disorder in the country (1, 2). An elevated serum uric acid level has been found associated with the development of gout, hypertension, coronary heart disease, and other components of the metabolic syndrome, resulting in significant economic burdens for both patients and society (3). Currently, allopurinol and febuxostat are frequently utilized in clinical treatment to lower uric acid levels. However, prolonged administration of these medications may lead to adverse effects, including hepatic and renal impairment (4). An increasing body of evidence suggests that polysaccharides have the potential activity to enhance the proliferation of harmful bacteria, restore the balance of intestinal microbiota, and facilitate the excretion of uric acid (5, 6). Additionally, polysaccharides have non-toxic side effects and high stability. Therefore, the development of polysaccharide resources to safely and effectively regulate uric acid metabolism has become the focus of the modern food industry in China.

The metabolism of dietary purines in the gastrointestinal tract results in the production of uric acid, with adenine and guanine being converted to adenosine monophosphate (AMP) and guanosine monophosphate (GMP). GMP undergoes conversion by nucleotidase and deamination by guanine deaminase to produce xanthine. Subsequently, XOD catalyzes the oxidation of hypoxanthine to xanthine, followed by the oxidation of xanthine to uric acid (7). Therefore, XOD plays a crucial role in the biosynthesis of uric acid. Metagenomic studies have revealed the involvement of intestinal microbiota in the purine degradation pathway leading to uric acid formation (8). Recent research has shown that metabolites of intestinal microbiota, such as SCFAs, can influence the level of uric acid in the serum and urine of HUA mice (9). In HUA mice, supplementation with acetic acid led to a significant reduction in serum uric acid level and inhibition of XOD activity (10). Overall, research indicates that the inhibition of XOD enzyme activity via the “microbiota-SCFAs” pathway may serve as a crucial mechanism for reducing uric acid levels.

China’s konjac output ranks first in the world, with an output value of approximately 25 to 30 billion yuan. KGM is the main component of konjac, consisting of a structural unit comprising a D-mannose and a D-glucose linked by a β-1,4 glycosidic bond. Research has demonstrated that KGM can effectively decrease serum uric acid levels in hyperuricemic rats (11, 12), yet the precise mechanism through which KGM modulates uric acid metabolism remains uncertain. Emerging evidence suggests that the biological activity of KGM is associated with its ability to regulate intestinal microbiota diversity and SCFA production (13). Our previous study found that KGM can significantly upregulate the abundance of SCFA-derived intestinal microbiota and promote the generation of fecal acetic acid, propionic acid, and butyric acid in diabetic rats (14, 15). Therefore, we hypothesized that KGM may improve uric acid metabolism by affecting intestinal microbiota and SCFA production. In this study, the effect of KGM on the microbiota of HUA patients was investigated using an anaerobic fermentation model *in vitro*. Furthermore, the XOD enzyme inhibitory activity of metabolites after fermentation of KGM was also evaluated. This study aims to explore the mechanism of KGM regulating uric acid metabolism based on “microbiota-SCFAs” and provide a theoretical basis for the targeted screening and mechanism research of uric acid-regulating active polysaccharides.

## 2 Materials and methods

### 2.1 Materials

KGM (KGM-M) with 90% purity was provided by Huaxianzi Konjac Products Co., Ltd. (Hubei, China). All other chemicals and reagents used were at least of analytical grade.

### 2.2 Fermentation of KGM *in vitro*

The fermentation of KGM *in vitro* was carried out according to the previous study (16). In brief, 4.5 g of yeast extract, 3.0 g of tryptone, 3.0 g of peptone, 0.5 g of mucin, 0.4 g of bile salt, 0.8 g of L-cysteine hydrochloride monohydrate, 4.5 g of NaCl, 2.5 g of KCl, 0.45 g of MgCl_2_·6H_2_O, 0.2 g of CaCl_2_·6H_2_O, 0.4 g of KH_2_PO_4_, 0.05 g of hemoglobin, and 1 mL of Tween 80 were dissolved in 1 L of distilled water to provide a basal nutrient medium after pH adjusting (to 7.0) and then autoclaved at 121°C for 20 min. Fecal samples were donated by three healthy volunteers (males, 20–30 years old) and three patients with HUA (males, 20–30 years old). The study was approved by the institutional review board of Panyu Central Hospital (IRB approval number: PYRC-2022-015), and written informed consent was obtained from all participants. Following collection, an equivalent mass of fecal samples obtained from healthy volunteers were mixed with sterilized physiological saline solution and centrifuged at 500 g for 5 min to obtain 10% healthy fecal suspension, which was regarded as the NOR group. Similarly, 10% HUA fecal suspension was obtained and named as the HUA group. Then, 1 mL of fecal supernatant was inoculated into a growth basal medium containing glucose or KGM (10 g/L), and healthy fecal samples were designated as the NOR-GLU and NOR-KGM groups, while HUA fecal samples were designated as the HUA-GLU and HUA-KGM groups. All samples were cultured in an anaerobic bag system at 37°C. After fermentation for 24 h, the samples were collected for further experiments.

### 2.3 The impact of fecal microbiota fermentation liquid on XOD enzyme activity

The inhibitory effect of fermentation samples on XOD activity was determined according to the following steps. Initially, 450 μL of 10 U/L XOD solution, 750 μL of fecal fermentation sample solution, and 525 μL of phosphate-buffered saline (PBS) solution were mixed uniformly. After incubating at 25°C for 15 min, 900 μL of 150 μmol/L of xanthine solution was added, followed by another incubation at 25°C for 30 min. The reaction was terminated by adding 375 μL of 1 mol/L of hydrochloric acid solution, and the absorbance was measured at 290 nm. For the positive control, the sample solution was replaced with different concentrations of allopurinol solution. For the sample control, the XOD solution was replaced with PBS. For the blank, the sample solution was replaced with an equal volume of PBS, and both the sample solution and XOD solution were replaced with PBS as the blank control. The inhibition rates of fermentation samples on XOD activity were calculated using the following formula (Equation 1).


(1) $$\mathrm{Inhibition}\;\mathrm{rate}\;\left(\%\right)=\left(1-\frac{A_{\mathrm{sample}}-A_{\mathrm{sample}\;\mathrm{control}}}{A_{\mathrm{blank}}-A_{\mathrm{blank}\;\mathrm{control}}}\right)\times 100$$

### 2.4 SCFAs analysis

SCFAs were measured according to previous research (14). After fermentation, 2 mL of the fermentation samples was taken and centrifuged at 4°C and 10,000 r/min for 5 min. A measure of 1 mL of the supernatant was collected, and then 0.1 mL of 50% H_2_SO_4_ and 1 mL of ether were added. The mixture was vortexed for 30 s to homogenize and then centrifuged again at 4°C and 10,000 r/min for 5 min. After being placed in a refrigerator at 4°C for 30 min, the upper layer was passed through a 0.45 μm filter membrane and collected in a brown sample vial for further analysis. The determination was carried out using a gas chromatograph equipped with a FID detector. The instrument conditions were as follows: FFAP flexible quartz capillary column measuring 30 m × 0.25 mm × 0.25 μm. The temperature program of the column was set at 100°C and then increased to 150°C at a rate of 5°C/min. Carrier gas was high-purity nitrogen with a purity of ≥99.999%. The carrier gas flow rate was 2 mL/min. The injector temperature was 250°C. The detector temperature (FID) was 280°C. The injection volume was 1 μL. Each sample was measured three times.

### 2.5 Intestinal microbiota analysis

#### 2.5.1 Extraction and amplification of total DNA from fermentation broth samples

After 24 h of fermentation, 1 mL of fermentation broth was used to extract the total bacterial DNA from the fermentation medium using the TIANamp Stool DNA Kit. The concentration and purity of the DNA samples were measured using the NanoDrop 2000C. Primers 338F and 806R were used to amplify the V3–V4 region of bacterial 16S rDNA for PCR amplification. The amplification procedure consisted of an initial denaturation at 95°C for 3 min, followed by 27 cycles of denaturation at 95°C for 30 s, annealing at 55°C for 30 s, and extension at 72°C for 30 s. The final extension was carried at 72°C for 10 min.

#### 2.5.2 Illumina MiSeq sequencing

The PCR products were recovered using a 2% agarose gel and purified using the AxyPrep DNA Gel Extraction Kit. They were then eluted with Tris-HCl and detected using 2% agarose gel electrophoresis. Quantification was performed using the QuantiFluor^™^-ST, following the standard operating procedures of the Illumina MiSeq platform. The purified amplified fragments were used to construct the library. Library construction included adapter ligation, removal of self-ligated fragments from the adapters using magnetic beads, enrichment of library templates by PCR amplification, magnetic bead recovery of PCR products, and sequencing on the Illumina MiSeq PE300 platform (Shanghai Majorbio Bio-pharm Technology Co., Ltd.), and data analysis was carried out through the www.i-sanger.com/ platform.

#### 2.5.3 Statistical analysis

GraphPad Prism 8.0 was applied for data analysis, and the results were presented as means ± SEM (*n* ≥ 3). A *p*-value of <0.05 indicates statistical significance, and the statistical significance is based on the one-way ANOVA procedure followed by Tukey’s test. The online platform of Majorbio I-Sanger Cloud was utilized for 16S sequence data processing. The correlation matrix between the microbiota and SCFAs was performed using Pearson’s test in OriginPro.

## 3 Results

### 3.1 Effect of KGM on intestinal microbiota

**Figure 1:**
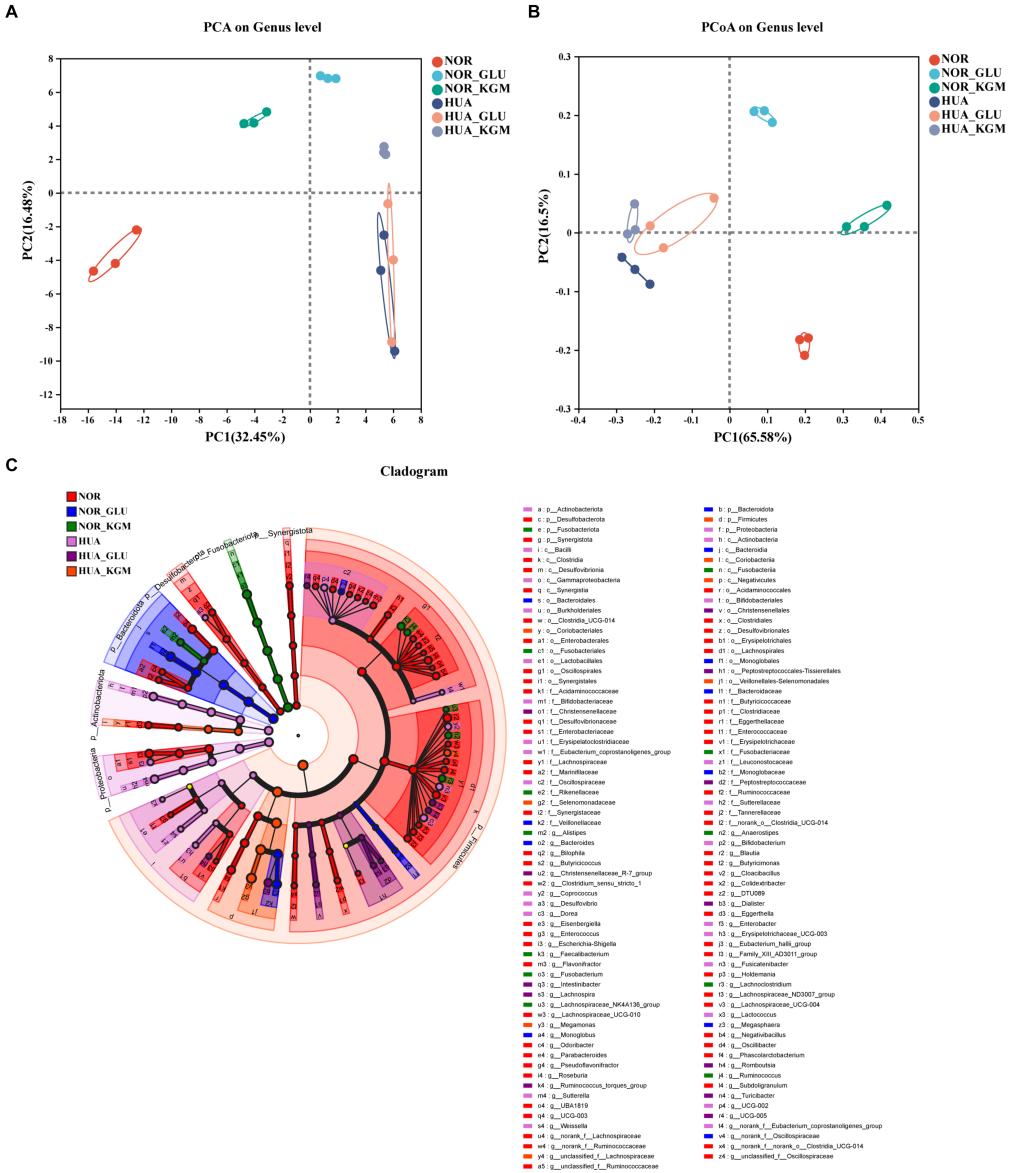
Effects of KGM on the structure and composition of gut microbiota are evaluated using beta-diversity analyses. PCoA based on the genus level. **(A)** PCA based on the genus level **(B)** and LEfSe based on the Bray–Curtis distance **(C)**.

The effects of KGM on the structure and composition of gut microbiota were evaluated using beta-diversity analyses (Figure 1). To display the differences of the genus in different samples, principal component analysis (PCA) was used to summarize factors mainly responsible for this difference. It was obvious that the different groups showed significantly different structures and compositions of gut microbiota. The treatment of KGM could shift the structure of HUA intestinal microbiota toward that of the NOR-KGM group as a result of PCA (Figure 1A), suggesting that KGM could improve the balance of HUA gut microbiota. Additionally, PCoA on genus level explained 65.58% of the total variance, indicating that HUA gut microbiota (HUA, HUA-GLU, and HUA-KGM) had different structures compared to those of healthy gut microbiota groups (NOR, NOR-GLU, and NOR-KGM). Furthermore, to determine the communities or species that explain the differences among the groups, LEfSe analysis was used to identify the biological characteristics representing the relationship among the groups. The circle radiating from inside to outside represents the classification level from the phylum level to the genus level. The diameter of the small circle represents the relative abundance of the classification at that level. As shown in Figure 1C, the representative taxa with significant differences among the groups were *Parabacteroides*, *Butyricicoccus*, *Clostridium*, and *Phascolarctobacterium* in the NOR group; *Bacteroides* and *Megasphaera* in the NOR-GLU group; *Anaerostipes*, *Ruminococcus*, *Lachnospiraceae*, and *Lachnoclostridium* in the NOR-KGM group; *Bifidobacterium*, *Enterobacter*, *Fusicatenibacter*, and *Coprococcus* in the HUA group; *Romboutsia*, *Intestinibacter*, *Ruminococcus*, *Lachnospira*, and *Turicibacter* in the HUA-GLU group; and *Megamonas* in the HUA-KGM group.

**Figure 2:**
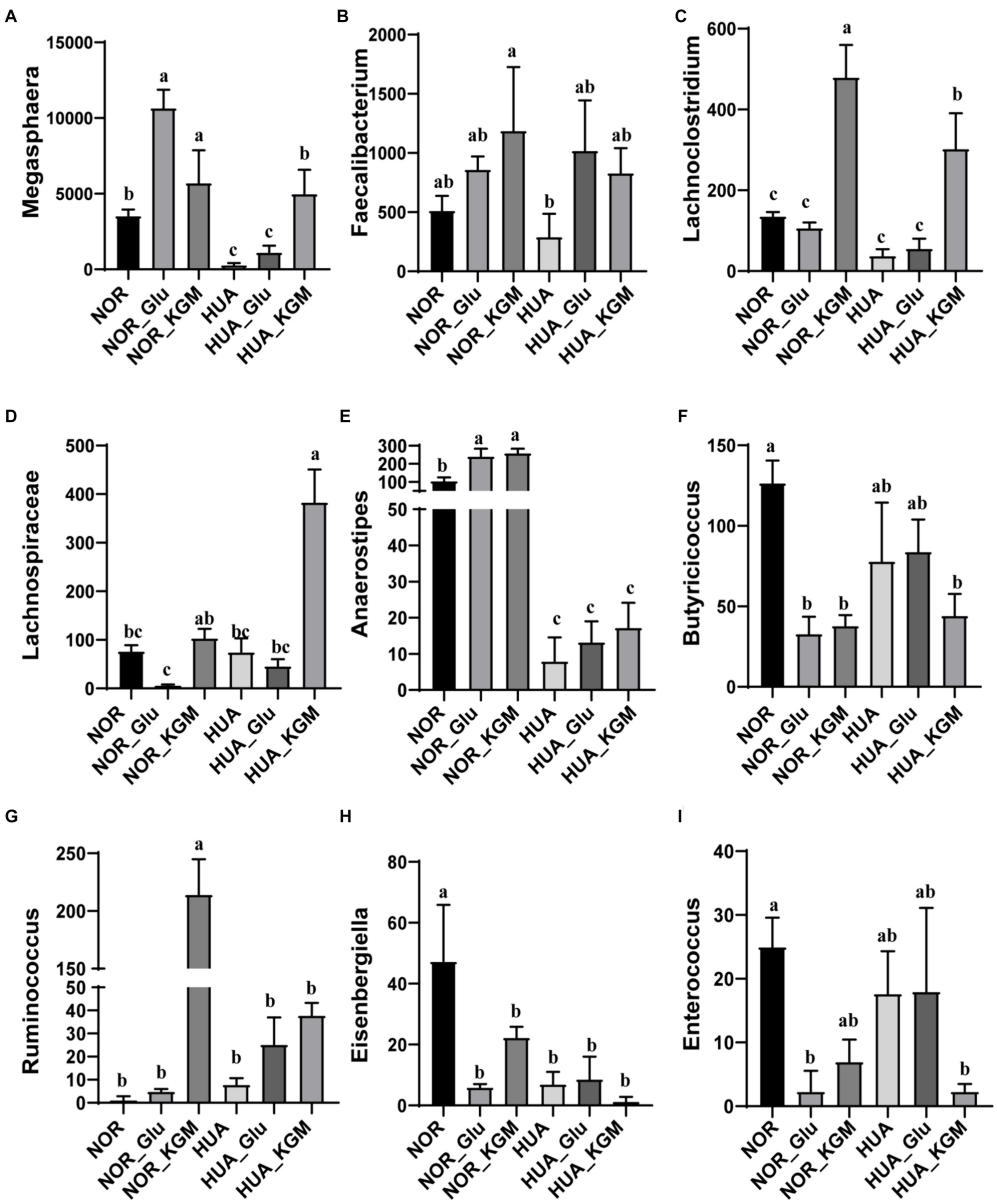
Relative abundance of gut microbiota at the genus level. The relative abundances of *Megasphaera* **(A)**, *Faecalibacterium* **(B)**, *Lachnoclostridium* **(C)**, *Lachnospiraceae* **(D)**, *Anaerostipes* **(E)**, *Butyricicoccus* **(F)**, *Ruminococcus* **(G)**, *Eisenbergiella* **(H)**, and *Enterococcus* **(I)**. The different letters indicate significant differences (*p* < 0.05) between groups, while the same letters indicate no significant difference (*p* > 0.05) between groups.

In Figure 2, the compositions of gut microbiota in both NOR and HUA groups were analyzed. In the NOR and its fermentation groups, *Megasphaera*, *Faecalibacterium*, *Lachnoclostridium*, *Anaerostipes*, *Butyricicoccus*, *Eisenbergiella*, and *Enterococcus* exhibited higher relative abundances than those in the HUA and its fermentation groups. Within the NOR groups, NOR-KGM increased the relative abundances of *Megasphaera*, *Faecalibacterium*, *Lachnoclostridium*, *Lachnospiraceae*, *Anaerostipes*, and *Ruminococcus*, while decreasing the relative abundances of *Butyricicoccus*, *Eisenbergiella*, and *Enterococcus* compared to the NOR group. Consistently, within the HUA groups, HUA-KGM can also increase the relative abundances of *Megasphaera*, *Faecalibacterium*, *Lachnoclostridium*, *Lachnospiraceae*, *Anaerostipes*, and *Ruminococcus*, while the relative abundances of *Butyricicoccus*, *Eisenbergiella*, and *Enterococcus* decreased compared to the HUA group. The glucose was regarded as a carbon source, which could partly improve the HUA gut microbiota by increasing the relative abundance of *Megasphaera*, *Faecalibacterium*, *Lachnoclostridium*, *Anaerostipes*, and *Ruminococcus*. However, the effect is much weaker when compared to the HUA group.

### 3.2 Analysis of SCFA content generated by KGM fermentation *in vitro*

**Figure 3:**
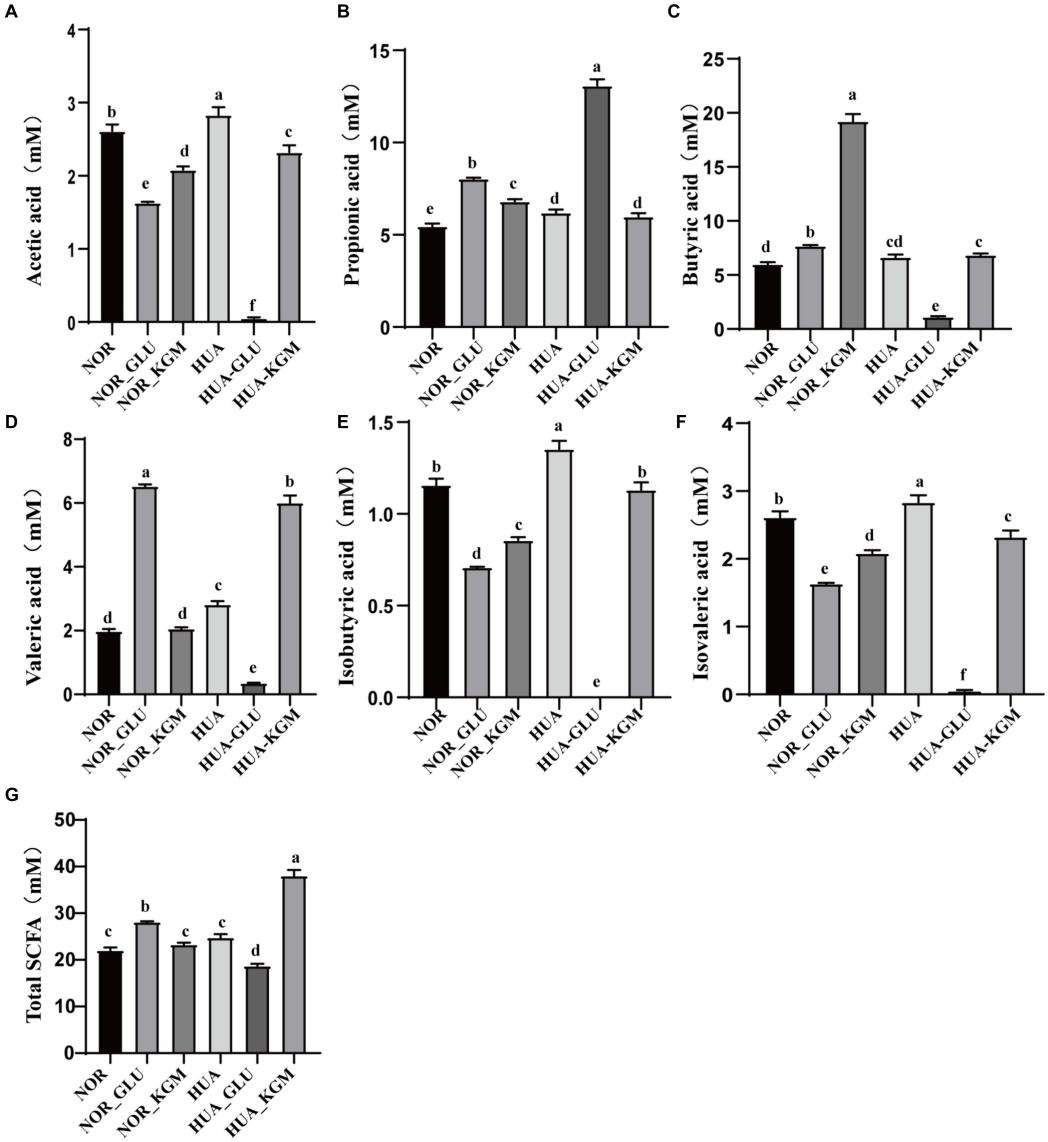
Levels of SCFAs in different groups after fermentation. Concentration of acetic acid **(A)**, propionic acid **(B)**, butyric acid **(C)**, valeric acid **(D)**, isobutyric acid **(E)**, isovaleric acid **(F)**, and total SCFAs **(G)** after fermentation. The different letters indicate that there are significant differences (*p* < 0.05) between every two groups, and the same letters indicate that there is no significant difference (*p* > 0.05) between every two groups.

The metabolites of intestinal microbiota are the key factors in the protective effects of dietary fiber in the host. SCFAs, including acetic acid, propionic acid, butyric acid, isobutyric acid, valeric acid, and isovaleric acid, are the main metabolites of KGM fermentation. In this study, SCFAs were measured to further investigate the fermentation characteristic of KGM and evaluate the potential health-promoting functions. As shown in Figure 3, acetic acid, propionic acid, butyric acid, valeric acid, isobutyric acid, and isovaleric acid were the main metabolites of KGM fermentation. NOR-KGM exhibited a similar increasing effect on acetic acid, propionic acid, butyric acid, isobutyric acid, and isovaleric acid levels in the NOR-GLU group when compared to the NOR group. Moreover, the concentration of butyric acid and valeric acid in the HUA-KGM group was significantly higher than that in the HUA group (*p* < 0.05), whereas acetic acid, isobutyric acid, and isovaleric acid in the HUA-KGM group were remarkably lower than that in the HUA group (*p* < 0.05). In addition, there was no significant difference in the total SCFAs level between the NOR (22.01 ± 0.61 mM) and NOR-KGM (23.28 ± 0.41 mM) groups. However, the total SCFAs level was significantly higher in the HUA-KGM group (38.02 ± 1.27 mM) than in the HUA (24.79 ± 0.71 mM) group. The total SCFAs level in the HUA-GLU group was remarkably lower than that in the HUA-KGM group. In particular, KGM could significantly promote the production of acetic acid, butyric acid, valeric acid, isobutyric acid, and isovaleric acid, which was higher than that in the HUA-GLU group. The results indicated that KGM may regulate the concentration of SCFAs, particularly by increasing the content of butyric acid and valeric acid and decreasing acetic acid, isobutyric acid, and isovaleric acid, by improving the composition of intestinal microbiota.

### 3.3 Inhibition rate of XOD enzyme activity of KGM *in vitro*

**Figure 4:**
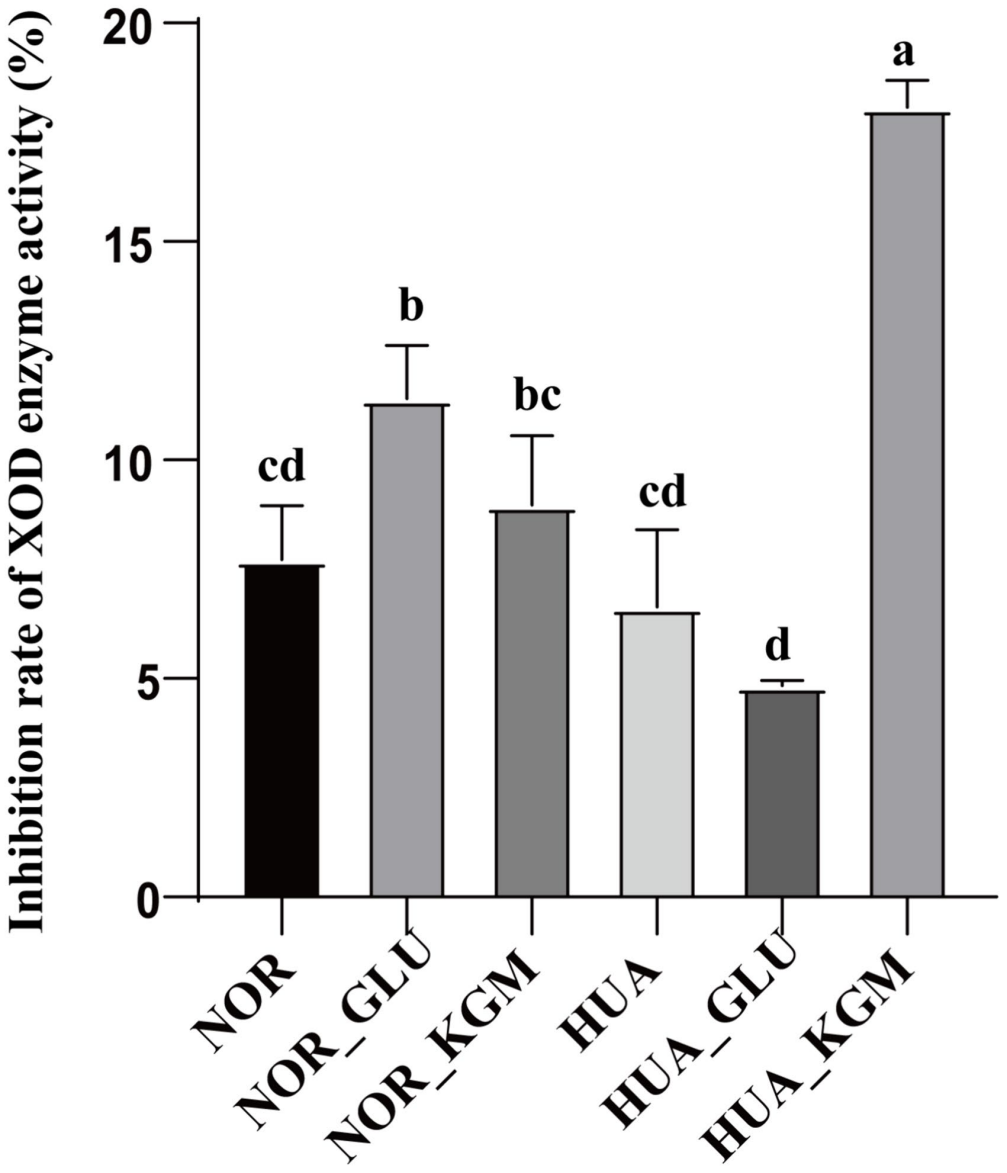
Effect of intestinal fermentation on XOD enzyme activity. The different letters indicate that there are significant differences (*p* < 0.05) between every two groups, and the same letters indicate that there is no significant difference (*p* > 0.05) between every two groups.

As described above, KGM could significantly increase the production of SCFAs. The inhibition rate of XOD activity of KGM fermentation was further evaluated *in vitro*. As shown in Figure 4, the XOD activity was decreased in the HUA group compared to the NOR group. In the NOR groups, the inhibitory rate of XOD activity in the NOR-KGM group was increased compared to the NOR group, indicating the potential mechanism of reducing the uric acid activity of KGM by suppressing XOD activity. Consistently, the XOD activity was significantly inhibited in the HUA-KGM group compared to that of the HUA group (*p* < 0.05). In addition, the inhibition rate of XOD enzyme activity in the HUA-GLU group was significantly lower compared to the HUA-KGM group. It suggested that the metabolites of KGM by intestinal microbiota may be the key factor contributing to the inhibitory effect on XOD activity.

**Figure 5:**
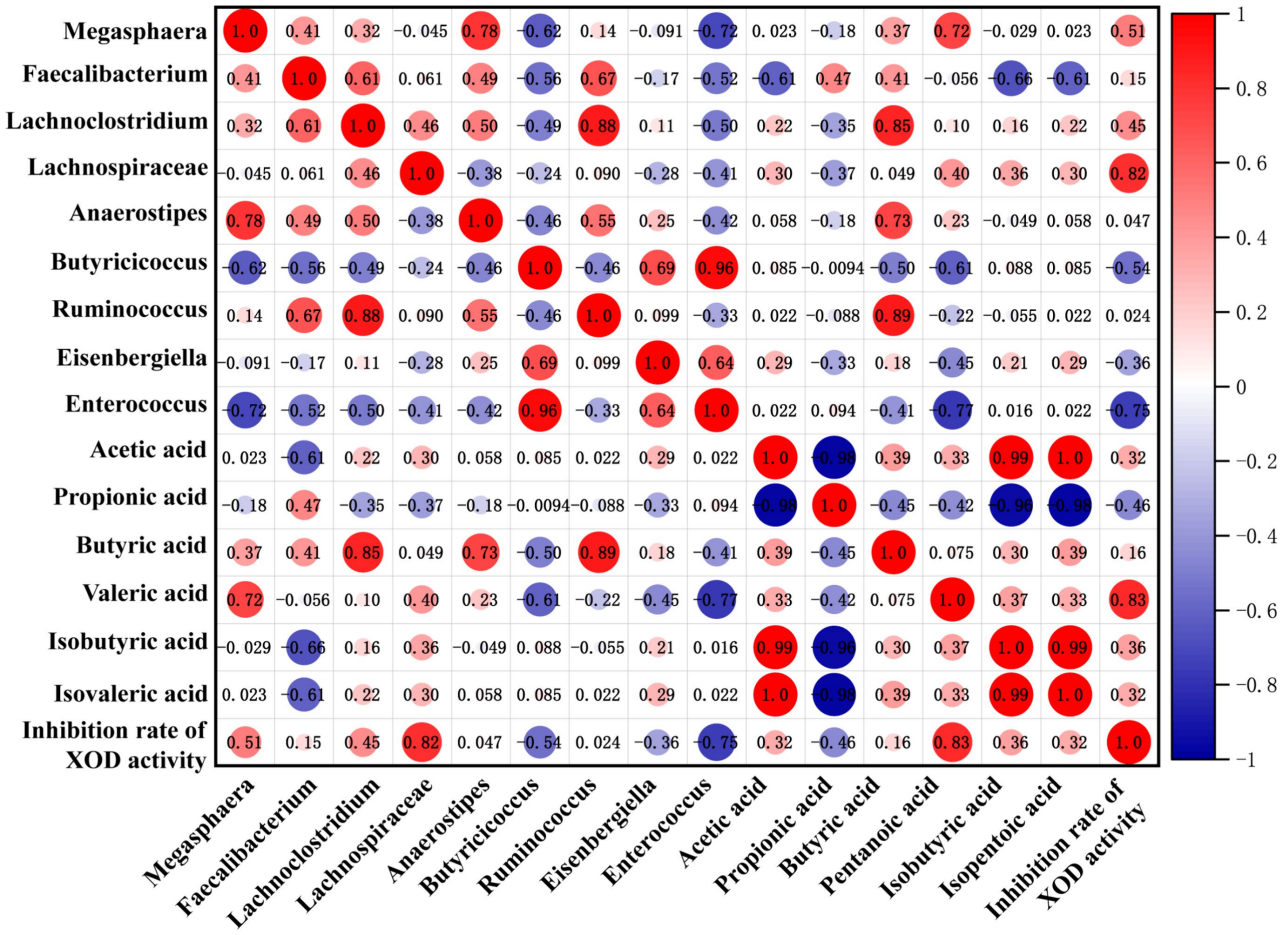
Correlation matrix among SCFA level, microbiota composition, and inhibition rate of XOD activity. A positive correlation is red, and the deeper the red, the higher the positive correlation. A negative correlation is blue, and the deeper the blue, the higher the negative correlation.

Based on the above results, it has been revealed that the regulation effect of KGM on SCFAs and SCFA-producing microbiota levels was consistent with its effect on XOD activity. Spearman correlation analysis was further used to visualize the relationship among SCFA level, microbiota composition, and inhibition rate of XOD activity. The results are shown in Figure 5, where the blue circle indicates a negative correlation, the red circle displays a positive correlation, and the size of the dots represents the magnitude of the correlation coefficient value. The larger and darker the dots, the stronger the correlation. In this study, the inhibition rate of XOD activity showed a strong negative correlation with the content of propionic acid, while it displayed a strong positive correlation with the concentration of valeric acid. This suggested that the inhibition of XOD activity by gut microbiota may relate to its regulation of SCFAs. Furthermore, there is a strong negative correlation between the relative abundance of *Butyricicoccus*, *Eisenbergiella*, and *Enterococcus* and the inhibition rate of XOD activity, while the relative abundance of *Megasphaera*, *Lachnoclostridium*, and *Lachnospiraceae* has a positive correlation with the inhibition rate of XOD activity, indicating that KGM may promote the production of metabolic product SCFAs by influencing the intestinal microbiota, thereby inhibiting the activity of XOD.

## 4 Discussion

The incidence of HUA gradually increases worldwide (17). Recently, the alteration of gut microbiota has been reported to be highly related to the initiation or progression of HUA (18). Furthermore, prebiotic supplementation has been regarded as a microbiome-targeted way to prevent diseases and improve host health (19). Thus, modulating the composition of gut microbiota by dietary intervention has been proposed as a new therapeutic target for the treatment of HUA. A previous study showed that KGM modulated the gut microbiota of HUA mice, which may contribute to the inhibition of XOD activity (12). In our previous study, it was found that KGM could regulate the gut microbiota and promote the production of SCFAs (14). Therefore, we speculated that the reshaping of intestinal microbiota by KGM to influence SCFA metabolism may be an important pathway for regulating uric acid metabolism. Based on the results of PCA and hierarchical cluster analysis, KGM could reverse the dysbiosis in the gut microbiota of the HUA group back to a healthier status. At the genus level, gut microbiota abundance of *Megasphaera*, *Faecalibacterium*, *Lachnoclostridium*, *Anaerostipes*, *Butyricicoccus*, *Eisenbergiella*, and *Enterococcus* in the NOR group showed a higher level than that of the HUA group. Usually, the composition of intestinal microbiota in adults is stable. The imbalance of gut microbiota may result in various diseases. It has been reported that the effect of microbiota on uric acid levels in mice may be related to the inhibitory activity of XOD (20). The differences in the composition of the gut microbiota between healthy individuals and HUA patients have been widely reported (18). The dysbiosis of gut microbiota mainly includes reduced microbial diversity. In addition, the depletion of *Megasphaera*, *Faecalibacterium*, *Lachnoclostridium*, *Lachnospiraceae*, *Anaerostipes*, and *Ruminococcus*, alongside the enrichment of *Eisenbergiella* and *Enterococcus*, observed in HUA mice, could lead to inflammation (21). Thus, the exploitation of directed microbiome-based therapies has been presented and highlighted in recent years. It has been reported that high levels of *Eisenbergiella* may contribute to chronic kidney disease, and experimental results have shown that *Eisenbergiella* is correlated with higher c-reactive protein levels (22). Moreover, *Enterococcus* was positively related to serum uric acid and urea nitrogen levels, which relate to the development of HUA nephropathy (21). Thus, the reduction of *Eisenbergiella* and *Enterococcus* could serve as a novel therapeutic target to improve HUA. In this study, the relative abundances of *Eisenbergiella* and *Enterococcus* at the genus level were elevated in the HUA group, but these levels were significantly reduced with the addition of KGM. This result suggests that KGM may have therapeutic potential for HUA, probably by reducing the levels of *Eisenbergiella* and *Enterococcus*.

On the other hand, *Lachnoclostridium* and *Lachnospiraceae* were regarded as important roles in the prevention of HUA. Recent studies showed that the *Lachnoclostridium* level was found to significantly decrease in HUA patients (23). In addition, *Lachnospiraceae*, a beneficial microbiota known for its anti-inflammatory properties, was found to decrease markedly in HUA mice (24). *Lachnoclostridium* is a Gram-positive genus that specializes in anaerobic fermentation to produce SCFAs such as butyric and acetic acids (25). These substances are crucial for renal protection due to their anti-inflammatory and antioxidant effects. It is known that *Lachnospiraceae* is one of the major producers of SCFAs, which can regulate inflammation and the immune system by producing butyric acid (26, 27). Thus, the modulation of *Lachnoclostridium* and *Lachnospiraceae* by KGM in HUA patients holds potential as a method for reducing disturbances in uric acid metabolism.

Furthermore, dietary polysaccharides have a protective effect not only through their direct impact on intestinal microbiota but also by the production of intestinal metabolites. Polysaccharides undergo fermentation by colonic microorganisms, leading to changes in gut microbiota composition and the generation of active metabolites, including SCFAs such as acetate, propionate, and butyrate (28). Studies have demonstrated that polysaccharides elicit a reduction in uric acid levels through the modulation of the intestinal environment and promotion of SCFA-producing bacterial populations (29). Previous research found that KGM can significantly reduce the UA level in the serum of diabetic rats and restore the glomerular structure (11). Our preliminary study showed that KGM can significantly increase the content of acetic acid, propionic acid, and butyric acid in the colon of diabetic rats, among which butyric acid production is the most significant (14). Recent research has indicated a significant decrease in the prevalence of SCFA-producing bacteria in HUA mice (19). Additionally, the production of butyric acid in the feces of individuals with a high level of uric acid was found to be significantly diminished (30), indicating a potential correlation between SCFAs and uric acid levels within the body. However, it is unclear whether KGM regulates UA metabolic enzyme activity by affecting the production of SCFAs through the intestinal microbiota. Our study findings provided evidence that KGM could significantly promote the proliferation of *Lachnoclostridium* and *Lachnospiraceae* at the genus level and increase the level of SCFAs in both NOR and HUA groups. Furthermore, KGM fermentation with high levels of SCFAs could significantly inhibit the activity of XOD. Thus, KGM is expected to be a potential microbiota-directed food for the modulation of the gut microbiota of HUA.

## 5 Conclusion

In conclusion, the inhibitory effect of KGM was investigated based on the gut microbiota in the present study. It was found that KGM could be metabolized into SCFAs by HUA gut microbiota. Furthermore, KGM could modulate the structure of HUA gut microbiota. At the genus level, KGM could decrease the relative abundances of *Butyricicoccus*, *Eisenbergiella*, and *Enterococcus*, while *Lachnoclostridium* and *Lachnospiraceae* in HUA gut microbiota were significantly increased by the addition of KGM. The metabolites of gut microbiota, such as SCFAs, might be responsible for the inhibition of XOD activity. Thus, KGM exhibited a superior probiotic function on the HUA gut microbiota, which is expected as a promising candidate for remodeling the HUA gut microbiota.

## Data Availability

The raw data supporting the conclusions of this article will be made available by the authors, without undue reservation.
